# Case Report: Clinical Experience With Avelumab in Patients With Metastatic Merkel Cell Carcinoma and Brain Metastases Treated in Europe

**DOI:** 10.3389/fonc.2021.672021

**Published:** 2021-05-28

**Authors:** Kate Fife, Pauline Tétu, Jessica Prabhakaran, Celeste Lebbé, Giovanni Grignani

**Affiliations:** ^1^ Oncology Department, Cambridge University Hospitals NHS Foundation Trust, Cambridge, United Kingdom; ^2^ Department of Dermatology, Assistance Publique des Hôpitaux de Paris Dermatology, Hôpital Saint-Louis, Paris, France; ^3^ University of Cambridge School of Clinical Medicine, Cambridge, United Kingdom; ^4^ AP-HP Dermatology and CIC, INSERM U976, Saint Louis Hospital, Université de Paris, Paris, France; ^5^ Candiolo Cancer Institute, FPO - IRCCS, Candiolo, Italy

**Keywords:** Merkel cell carcinoma, brain metastases, avelumab, immunotherapy, stereotactic radiosurgery

## Abstract

Merkel cell carcinoma (MCC) is a rare and aggressive skin cancer that can metastasize rapidly. In patients with metastatic MCC (mMCC), brain metastases are uncommon but are associated with poor prognosis; furthermore, there is limited published literature regarding treatment of these patients, and no specific regimens are currently recommended by guidelines. Avelumab, an anti–programmed death ligand 1 monoclonal antibody, was the first approved treatment for patients with mMCC. Here, we present 4 cases of patients with mMCC and brain metastases treated with avelumab. Patient age ranged from 48 to 70 years, and all patients received avelumab as second-line therapy following disease progression with platinum-based chemotherapy. Patient cases 1 and 2 received avelumab alone and experienced rapid disease progression according to Response Evaluation Criteria in Solid Tumors version 1.1 (RECIST 1.1). In patient case 3, avelumab alone resulted in a prolonged complete response by RECIST 1.1 of 1 brain metastasis and partial response by RECIST 1.1 of a second brain metastasis. After 11 months of avelumab treatment, the patient received concurrent stereotactic radiosurgery that resulted in complete response of the second metastasis. Patient case 4 achieved a partial response by RECIST 1.1 with avelumab plus stereotactic radiosurgery. These results suggest that avelumab followed by radiotherapy or with concurrent radiotherapy may be an effective treatment option for patients with mMCC and brain metastases.

## Introduction

Merkel cell carcinoma (MCC) is a rare neuroendocrine tumor associated with UV radiation exposure, clonal integration of the Merkel cell polyomavirus, and immunosuppression (1). MCC commonly occurs in sun-exposed areas of the body such as the head and neck region (1). However, in approximately 4-5% of all patients and 28-40% of those with clinically detectable nodal disease, the primary lesion cannot be identified; these cases are associated with a more favorable prognosis (1–3).

MCC is an aggressive disease that can metastasize early (4); at diagnosis, approximately 26% and 8% of patients have nodal and distant metastatic MCC (mMCC), respectively (2). Metastases usually arise in the lymph nodes, skin, bone, lung, or liver (4). Brain metastases are less common and occur in approximately 7-13% of patients with distant mMCC (4–6). In patients with MCC, the occurrence of brain metastases is associated with a poor prognosis, with a median overall survival (OS) of approximately 2 years without neurosurgery (6). There are limited published data on patients with mMCC and brain metastases, and many trials in MCC exclude this subset of patients (6). Furthermore, there are no treatment options recommended by guidelines specifically for patients with mMCC and brain metastases (4, 7); however, a possible survival benefit has been suggested for patients who receive surgery or radiotherapy (6).

Avelumab, an anti–programmed death ligand 1 (PD-L1) monoclonal antibody, became the first approved treatment for mMCC based on promising results in the phase 2 JAVELIN Merkel 200 trial (8, 9). Patients with brain metastasis were excluded from this trial. Prior to approval, avelumab showed clinical benefit in a real-world setting in patients with mMCC and limited treatment options (including immunocompromised patients and those with treated brain metastases) in the global expanded access program (10).

Here, we report the clinical experiences of 4 patients with mMCC and brain metastases treated with avelumab in Europe.

## Patient Cases

The patient cases are summarized in **Table 1**.

**Table 1:** Summary of patient cases.

	Patient case 1	Patient case 2	Patient case 3	Patient case 4
**Sex**	Female	Female	Female	Male
**Age, years**	70	48	67	66
**Comorbidities**	Hyperuricemia, arterial hypertension, hypothyroidism (treated with hormone replacement therapy)	None	None	High blood pressure, depression, dyslipidemia
**Date of diagnosis of mMCC**	October 2016	February 2017	January 2017	March 2016 (confirmed in June 2017)
**Site of primary lesion**	Left thigh	Unknown	Unknown	Unknown
**Site of metastases at baseline**	Inguinal and paraaortic/iliac nodes	Right lumboaortic, retrocrural, inguinal, and common external iliac nodes	Left axilla, neck, and supraclavicular nodes	Retroclavicular, retropectoral, and right axillary nodes
**Treatment before avelumab**	Cisplatin + etoposide	Cisplatin + etoposide	Carboplatin + etoposide, surgery	Surgery, radiotherapy, carboplatin + etoposide
**Date brain metastases identified**	October 2, 2017	August 18, 2017	May 9, 2018	December 31, 2017
**No. of brain metastases**	1	1	2	1
**Symptoms associated with brain metastases**	None	None	None	None
**ECOG PS at start of avelumab treatment**	1	2	0	1
**Date of first avelumab dose**	October 4, 2017	August 30, 2017	June 26, 2018	December 26, 2017
**Avelumab treatment**	2L monotherapy	2L monotherapy	2L + SRS	2L with concurrent SRS
**Approximate duration of avelumab treatment at last follow-up, months**	1	2	17	15
**Best response to avelumab per RECIST 1.1**	Progressive disease	Stable disease	Complete response in 1 metastasis; partial response in 1 metastasis*	Partial response^†^
**Toxicity associated with avelumab**	None	None	Sinus node disease probably related to avelumab treatment	None
**Subsequent treatment**	Palliative care	Radiotherapy + chemotherapy, palliative care	Radiotherapy	Nivolumab with concurrent SRS
**Avelumab treatment ongoing at last follow-up**	No	No	No	No
**Vital status at last follow-up (date)**	Died	Died	Alive (November 2020)	Alive (March 2021)

2L, second line; mMCC, metastatic Merkel cell carcinoma; RECIST 1.1, Response Evaluation Criteria in Solid Tumors version 1.1; SRS, stereotactic radiosurgery.

*Patient achieved a partial response in 1 brain metastasis with avelumab, this metastasis then progressed (not confirmed by RECIST 1.1) and the patient received SRS with avelumab and later achieved a complete response by RECIST 1.1 in that metastasis. ^†^Patient achieved a complete response following subsequent nivolumab treatment.

### Patient Case 1

A 70-year-old woman from Italy with hyperuricemia, arterial hypertension, and hypothyroidism presented with pain and a growing thigh mass and was diagnosed with mMCC of the left thigh and multiple nodal metastases (inguinal and paraaortic/iliac lymph nodes) in October 2016. The patient had a family history of cancer (gastric cancer and leukemia) and had previously received a bilateral total knee prosthesis and undergone a right saphenectomy. Between November 2016 and March 2017, the patient received 6 cycles of chemotherapy (cisplatin 25 mg/m^2^ plus etoposide 100 mg/m^2^ on days 1-3 every 21 days), with no relevant acute toxicity.

**Figure 1:**
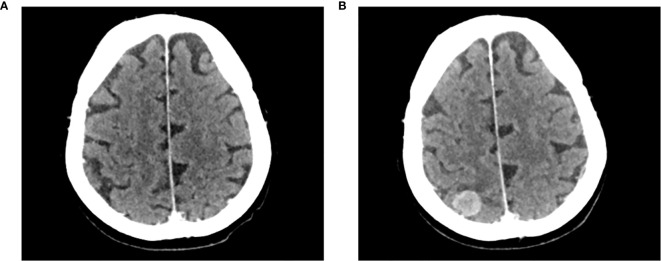
Development of an asymptomatic brain metastasis in patient case 1. Computed tomography brain scans of patient case 1 prior to initiating treatment with avelumab **(A)** on August 16, 2017, and **(B)** on October 2, 2017.

On April 5, 2017, the patient presented with worsened left leg edema, and a subsequent whole-body positron emission tomography (PET) scan showed an increase in the number of metastases of fluorodeoxyglucose uptake compared with the previous PET scan in November 2016, indicating disease progression. On April 12, 2017, a whole-body computed tomography (CT) scan, including the brain, showed progressive disease (PD) by Response Evaluation Criteria in Solid Tumors version 1.1 (RECIST 1.1) with increased size of the left leg edema and left inguinal lymph node metastases. The patient subsequently received 3 cycles of chemotherapy (VAC regimen: vincristine 2 mg plus doxorubicin 50 mg/m^2^ plus cyclophosphamide 1000 mg/m^2^ every 21 days); however, the patient experienced toxicity and further PD with an increase in the dimensions and number of abdominal lymph node and pelvic metastases, and cutaneous and subcutaneous nodules. At this time, the patient also developed 1 asymptomatic brain metastasis (largest diameter, 18 mm on October 2, 2017; **Figure 1**). Due to the asymptomatic nature of the brain metastasis, neurosurgery was not considered.

The patient began treatment with avelumab (10 mg/kg intravenously every 2 weeks [Q2W]) on October 4, 2017; at this time, the patient had an Eastern Cooperative Oncology Group performance status (ECOG PS) of 1. Avelumab was well tolerated, with no adverse events reported, and the patient had reduced pain during the first 2-3 infusions; however, after a total of 4 infusions, the treatment was stopped on November 14, 2017, because of further local (left thigh cutaneous lesion) and systemic (brain metastasis) clinical and radiological progression. The patient then received palliative care and died approximately 4 weeks later.

### Patient Case 2

A 48-year-old woman from Italy with a family history of cancer (biliary tract) was diagnosed with mMCC of the right inguinal region with nodal (right lumboaortic, retrocrural, inguinal, and common external iliac) and mammary gland metastases in February 2017. The patient had moderate pain which was controlled with paracetamol. No primary cutaneous lesion was identified. Immunohistochemical analysis showed expression of synaptophysin, cytokeratin (CK) 20, and high levels of Ki67 (70%). Between March and July 2017, the patient underwent 6 cycles of chemotherapy (cisplatin 30 mg/m^2^ plus etoposide 100 mg/m^2^ on days 1-3 every 21 days), with no relevant toxicity.

On August 18, 2017, a CT scan showed PD by RECIST 1.1 of the nodal, lung, and bone regions and the appearance of an asymptomatic meningeal metastasis close to the ethmoid region. Due to the unusual location of the brain metastasis, the patient was considered too high risk for neurosurgery. The patient was enrolled in the avelumab global expanded access program on August 30, 2017 and began treatment with avelumab (10 mg/kg intravenously Q2W); at this time, the patient had an ECOG PS of 2. Avelumab was well tolerated, with a reduction in pain and no adverse events reported; however, despite initial stable disease, the patient experienced rapid PD (including the meningeal metastasis) by RECIST 1.1 after 5 infusions. Avelumab treatment was subsequently stopped on October 25, 2017. The patient then underwent radiotherapy (39 Gy in 13 fractions) for the inguinal metastasis and received subsequent chemotherapy (oral etoposide 50 mg on days 1-14 every 28 days); however, by February 2018, the patient had experienced further PD. The patient was then referred to palliative care and died approximately 5 months later.

### Patient Case 3

A 67-year-old British woman with a history of congenital jejunal diverticular bleeding (treated with resection) was diagnosed with mMCC in January 2017, after presenting with a large left axillary mass and lymphoedema; no primary site was identified. The patient did not have a family history of cancer. Immunohistochemical analysis showed expression of synaptophysin, CD56, CK7, and CK20 and no expression of thyroid transcription factor 1, calcitonin, or CDX-2. A PET-CT scan revealed high fluorodeoxyglucose uptake in the left axilla (largest node, 39 mm), neck, and supraclavicular nodes. No primary skin lesion was identified. The patient received 4 cycles of neoadjuvant chemotherapy (carboplatin AUC 5 plus etoposide 120 mg/m^2^ on days 1-3 every 28 days) and experienced a partial response by RECIST 1.1 after 3 months (July 2017); however, the patient subsequently experienced PD. In September 2017, the patient underwent surgical dissections of the level I-V nodal regions in the left side of the neck and a level I-III axillary node. Following surgery, a PET-CT scan showed no fluorodeoxyglucose-avid disease. The patient declined adjuvant radiotherapy due to the risk of the left arm lymphoedema worsening.

**Figure 2:**
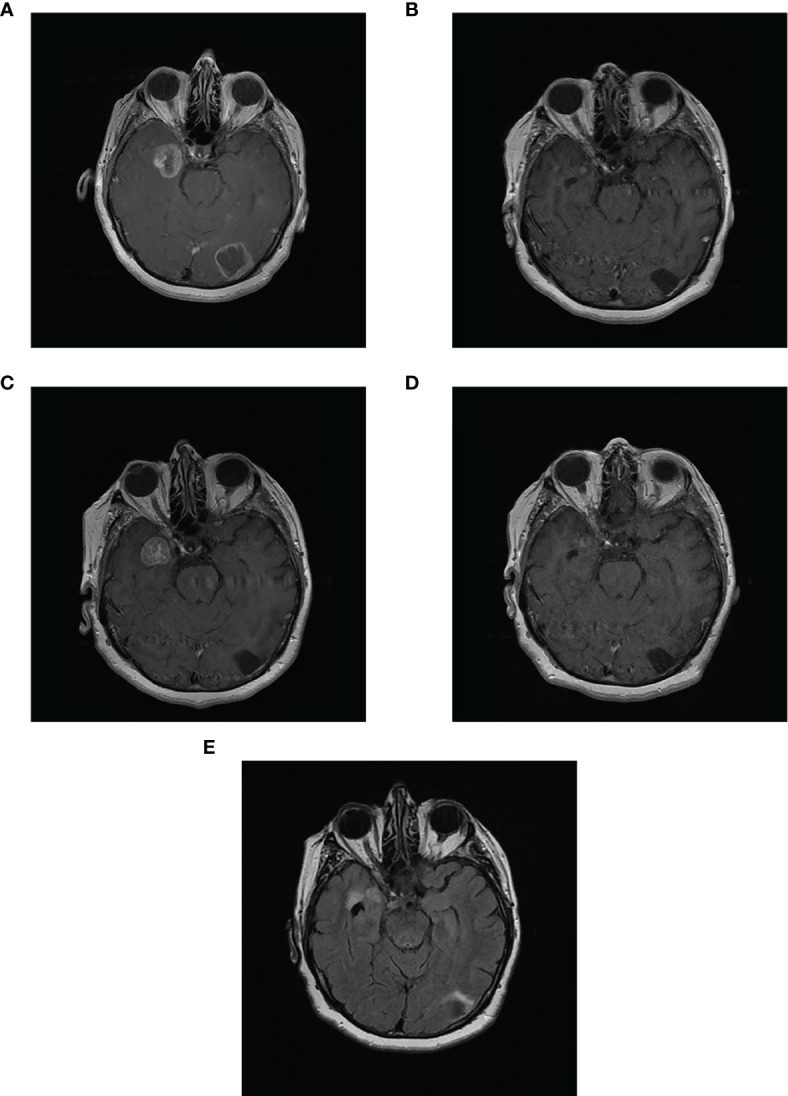
Complete response of 2 asymptomatic brain metastases in patient case 3. Magnetic resonance imaging scans of patient case 3: **(A)** with 2 brain metastases in the left occipital lobe and right temporal lobe prior to starting avelumab (May 9, 2018); **(B)** Complete response in occipital lobe and partial response in temporal lobe metastases after 8 months of avelumab (January 21, 2019); **(C)** PD in temporal lobe metastasis after 10 months of avelumab (April 29, 2019; patient began concurrent SRS on May 31, 2019); **(D)** substantial decrease in size of temporal lobe metastasis after SRS and 15 months of avelumab (September 4, 2019); **(E)** Complete response in both metastases (June 16, 2020; avelumab treatment stopped on February 24, 2020).

In April 2018, a PET scan showed PD by RECIST 1.1 in the left supraclavicular (30-mm node) and axilla regions, and a brain magnetic resonance imaging (MRI) scan on May 9, 2018, showed the presence of 2 asymptomatic brain metastases in the right medial temporal lobe adjacent to the optic chiasm (29 mm×25 mm) and left occipital lobe (33 mm×31 mm; **Figure 2A**). Because of the location of the temporal lobe metastasis, it was not possible to safely undertake neurosurgery or stereotactic radiosurgery (SRS).

On June 26, 2018, the patient began avelumab treatment (10 mg/kg intravenously Q2W); at this time, the patient had an ECOG PS of 0. After 4 months of avelumab treatment, an MRI scan on October 17, 2018, showed a complete response by RECIST 1.1 of the occipital metastasis and a partial response by RECIST 1.1 of the temporal lobe metastasis (reduced to 21 mm). On January 21, 2019, an MRI scan showed a substantial decrease in the size of the temporal lobe metastasis to 5 mm×3 mm; the left occipital lobe metastasis was cystic with no enhancement (**Table 2**; **Figure 2B**).

**Table 2:** Timeline of treatments received and brain metastases size in patient case 3.

Date of MRI scan; treatment received	Dimensions of brain metastases, mm	
	Right temporal lobe	Left occipital lobe
May 9, 2018; prior to starting avelumab	29×25	33×31
**June 26, 2018; started avelumab (10 mg/kg every 2 weeks)**		
July 23, 2018; 1 month of avelumab	27×19	32×35
October 17, 2018; 4 months of avelumab	21×9	NA (focal volume loss and no residual enhancement)
January 21, 2019; 8 months of avelumab	5×3	NA
April 29, 2019; 10 months of avelumab	21 (maximum transverse dimension)	NA
**May 14, 2019 to June 23, 2019; avelumab paused** **May 31, 2019 to June 5, 2019; received SRS (25.5 Gy in 3 fractions over a week)**		
September 4, 2019; 14 months of avelumab*	8 (maximum transverse dimension)	NA
January 25, 2020; 16 months of avelumab^†^	NA (no residual enhancement)	NA
June 16, 2020	NA (no intracranial mass or abnormal contrast enhancement)	NA
October 28, 2020	NA (focal volume loss; no mass or enhancement)	NA (focal volume loss; no mass or enhancement)

MRI, magnetic resonance imaging; NA, not applicable; SRS, stereotactic radiosurgery.

*The patient stopped avelumab treatment on November 25, 2019, and received external beam radiotherapy to the bilateral neck region on December 2, 2019, to improve localized control of the disease progression [40 Gy in 15 fractions in 3 weeks (1 fraction per day)].

^†^The patient resumed avelumab treatment on January 13, 2020, for 2 months (last avelumab dose February 24, 2020).

On April 29, 2019, a brain MRI scan showed that the anterior medial part of the right temporal lobe metastasis was larger (maximum transverse dimension of 21 mm) and surrounded by increased white matter edema (**Figure 2C**). There was no change in the area of cystic encephalomalacia in the left occipital lobe compared with January 2019. Given this observed progression in the right temporal metastasis, avelumab treatment was paused (from May 14 to June 23, 2019), and the patient began treatment with fractionated SRS for this metastasis from May 31 to June 5, 2019 (25.5 Gy in 3 fractions).

On September 4, 2019, an MRI scan showed the right temporal metastasis had reduced in size (maximum transverse dimension of 8 mm; **Figure 2D**). In October 2019, clinical evaluation showed enlargement of the left-sided neck mass, and a PET scan showed a left-sided lower neck mass of 30 mm, fluorodeoxyglucose uptake in the small left upper cervical node, and a new right upper cervical node. The patient stopped avelumab treatment on November 25, 2019 and received external beam radiotherapy to the bilateral neck region on December 2, 2019, to improve localized control of the disease progression (40 Gy in 15 fractions in 3 weeks [1 fraction per day]). The patient then resumed avelumab treatment on January 13, 2020, for 2 cycles (last dose February 24, 2020). On January 25, 2020, an MRI scan showed a complete response by RECIST 1.1 of the brain metastases with no residual enhancement of the right temporal lobe. On February 24, 2020, a CT scan showed a complete response by RECIST 1.1 in all metastases including in the extracranial regions (i.e., the neck).

In March 2020, the patient had experienced 3 recent drop attacks; cardiac investigations, including an implantable heart monitor, detected sinus pauses of ≤28 seconds due to sinus node disease. Cardiac MRI and echocardiogram were normal, as were troponin, B-type natriuretic peptide, and creatine kinase concentrations; the patient had no risk factors for cardiac disease. Therefore, it was likely that this adverse event was related to avelumab treatment. The patient received a dual chamber pacemaker on April 7, 2020. MRI scans on June 16 (**Figure 2E**) and October 28, 2020 showed volume loss and no enhancement of the brain metastases. At last follow-up, on November 28, 2020, a body CT scan showed no recurrence of metastases. The patient is delighted with the outcome of her treatment and, having been off treatment for over a year, continues to be able to maintain an active lifestyle.

### Patient Case 4

A 66-year-old man from Algeria but treated in France with a history of high blood pressure, depression, and dyslipidemia presented with an asymptomatic, left parotid tumefaction in July 2015. He had no family history of cancer and no relevant prior interventions. An exofacial left parotidectomy, including a biopsy of areas II and IV, was performed in March 2016. This biopsy showed a high-grade neuroendocrine carcinoma that was considered to be a potential metastasis. A whole-body PET scan and a cervical, thoracic, and abdominal CT scan had negative findings at the time, and no primary tumor site was found. Adjuvant radiotherapy (60 Gy in 30 fractions) was performed on the surgical area from May to July 2016. In June 2017, the patient presented with right axillary adenopathy, and a whole-body PET scan and a cervical, thoracic, and abdominal CT scan found substantial retroclavicular, retropectoral, and right axillary node metastases. A biopsy of the right axillary node was used to diagnose MCC.

From August 7 to October 18, 2017, the patient received 4 cycles of chemotherapy (carboplatin AUC 5 plus etoposide 100 mg/m^2^ on days 1-3 every 28 days). In November 2017, a CT scan found node, muscle, and pararectal disease progression. In December 2017, the patient presented with pain and dysesthesia of the right arm due to right axillary adenopathy. Second-line avelumab treatment (10 mg/kg Q2W) was started on December 26, 2017, and was well tolerated (no adverse events were reported and the pain and dysesthesia of the right arm rapidly improved); at this time, the patient had an ECOG PS of 1. On December 31, 2017, a brain MRI scan showed an asymptomatic left cerebellar metastasis measuring <1 cm; neurosurgery was not considered to be necessary, and the patient received concurrent SRS (20 Gy in 1 fraction) of the cerebellar metastasis and began palliative radiotherapy (30 Gy in 10 fractions) of the right axillary mass.

In March 2018, a CT scan showed an extracranial partial response according to RECIST 1.1 (68% decrease), and an MRI scan showed regression of the cerebellar metastasis. Avelumab was discontinued in January 2019 after 25 cycles (13 months) of treatment, due to the persistent partial response and the difficulties for the patient to travel to receive avelumab.

Six months after avelumab treatment was stopped (July 2019), a brain MRI scan showed recurrence of the cerebral metastasis, justifying resumption of avelumab and 1 dose of SRS (16 Gy in 1 fraction). At this time, the patient was still experiencing an extracranial partial response. After 2 months, the patient switched from avelumab to nivolumab (anti–programmed death 1 [PD-1]; 480 mg IV every month) to reduce the patient’s travel burden. In July 2020, an MRI scan showed that the patient remained in extracranial partial response and had also achieved partial response of the brain metastases; however, radionecrosis of the previously irradiated cerebral metastasis was found, and the patient was subsequently treated with corticosteroids 0.5 mg/kg with good resolution. At last follow-up (March 2021), an MRI scan showed both intracranial and extracranial complete response by RECIST 1.1.

## Discussion

MCC is a rare tumor, but incidences have increased in recent years with approximately 5000 new cases of MCC annually in the US and Europe (11, 12). Patients with mMCC have a poor prognosis, with historical 5-year OS rates of 35% and 14% for nodal and distant disease, respectively (2). Current guidelines for the treatment of mMCC recommend enrollment in a clinical trial or systemic therapy with an anti–PD-1/PD-L1 antibody (4).

In 2017, avelumab became the first approved treatment for mMCC based on the results of the JAVELIN Merkel 200 trial (8, 9). Initially, this approval was based on primary analysis results from a cohort of patients with mMCC who received avelumab as second-line or later treatment after disease progression with chemotherapy (part A) (8) and preliminary data from a subset of patients who received avelumab as first-line treatment, which was initiated subsequently (part B) (9). After 3 years of follow-up from part A of the trial (N=88), the objective response rate was 33.0% (95% CI, 23.3-43.8) and median duration of response was 40.5 months (95% CI, 18.0-not estimable). Median progression-free survival and OS was 2.7 months (95% CI, 1.4-6.9) and 12.6 months (95% CI, 7.5-17.1), respectively (8, 13). After ≥15 months of follow-up in part B (N=116), the objective response rate was 39.7% (95% CI, 30.7-49.2), and median duration of response was 18.2 months (95% CI, 11.3-not estimable). Median progression-free survival and OS was 4.1 months (95% CI, 1.4-6.1) and 20.3 months (95% CI, 12.4-not estimable), respectively (14). The JAVELIN Merkel 200 trial excluded patients with active central nervous system metastases (8), and limited data are available for the treatment of these patients.

In the cases reported here, patient age ranged from 48 to 70 years, 3 of the 4 patients were female, and 2 had comorbidities. All patients had asymptomatic brain metastases and received avelumab as second-line therapy following disease progression with platinum-based chemotherapy. Avelumab treatment was well tolerated in 3 patients; however, 1 patient (case 3) developed sinus node disease that was likely related to avelumab treatment.

MCC is an aggressive cancer, and disease progression and development of metastases can occur early (4). In the cases reported here, slower tumor growth and the use of SRS appeared to correlate with better response to subsequent avelumab treatment. Two of the 4 patients experienced rapid progression, with brain metastases identified approximately 6 months (patient 2) and 12 months (patient 1) after initial mMCC diagnosis, and further disease progression with avelumab. In the remaining 2 patients, progression appeared more gradual, with brain metastases diagnosed approximately 16 months (patient 3) and 22 months (patient 4) after initial diagnosis. With avelumab treatment alone, patient 3 experienced a complete response in 1 brain metastasis and partial response in a second brain metastasis; the second metastasis then progressed, but further treatment with avelumab and SRS led to a complete response. Patient 4 experienced a partial response with avelumab plus concurrent SRS, and subsequently achieved a complete response after switching to nivolumab treatment. Patient ECOG PS at the time of starting avelumab treatment did not appear to be associated with a better response.

Although no treatment options are recommended by guidelines for patients with mMCC and brain metastases (4, 7), radiotherapy is commonly used and has been associated with a survival benefit (6). Furthermore, SRS with concurrent immunotherapy is recommended by the European Society for Medical Oncology for patients with melanoma and brain metastases (15). In a recent study of 262 patients with melanoma and brain metastases, radiotherapy combined with either immunotherapy or targeted therapy was associated with a significantly reduced risk of death *vs* systemic therapy (median OS 16.8 *vs* 6.9 months, respectively) (16). In the cases reported here, SRS administered 11 months after starting avelumab treatment (patient 3) or at the same time as starting avelumab (patient 4) resulted in a complete response and a partial response, respectively, and both patients were alive at last follow-up. Neurosurgery has also been associated with prolonged survival in patients with mMCC and brain metastases (6); however, in the cases reported here, patients did not undergo neurosurgery due to the asymptomatic nature of the metastases and their locations being considered too high risk for resection. Additionally, combining immunotherapy and SRS does not appear to increase toxicity compared with SRS alone (17).

Furthermore, patients with nodal mMCC and no known primary tumor location have been shown to have longer OS compared with those with a known primary tumor site (2, 18). This may be due to a more active immune response in some patients that is able to eliminate the primary tumor (18). For the 2 patients with the longest OS in this series, cases 3 and 4, no primary tumor was identified. However, the dataset is small, and immune markers were not analyzed; therefore, further research is needed to investigate the potential mechanisms involved.

In summary, published data for patients with mMCC and brain metastases are limited. We report the clinical experiences of 4 patients with mMCC and brain metastases treated in Europe with avelumab after prior disease progression with chemotherapy. In this small series, we show that avelumab can have intracerebral and systemic activity and, when combined with radiotherapy (both brain SRS and radiotherapy to other sites), can produce lasting disease control. Although the optimal timing of SRS administration warrants further investigation, the combination of SRS and avelumab, along with more gradual disease progression, appeared to be associated with improved survival. Further research into potential predictors of response and prolonged survival in this subset of patients is needed. Although clinical trials in this small subset of patients are unlikely to be feasible, prospective data on the use of SRS plus immunotherapy in patients with mMCC and brain metastases along with data from trials investigating this combination in patients with other more common tumor types will provide further insight into this treatment strategy. Overall, our findings indicate that the use of SRS with immunotherapy may be an effective treatment option for patients with mMCC and brain metastases.

## Data Availability Statement

The original contributions presented in the study are included in the article/supplementary material. Further inquiries can be directed to the corresponding author.

## Ethics Statement

Ethical review and approval was not required for the study on human participants in accordance with the local legislation and institutional requirements. The patients/participants provided their written informed consent to participate in this study. Written informed consent was obtained from the individual(s) for the publication of any potentially identifiable images or data included in this article.

## Author Contributions

All authors contributed to data collection and interpretation and writing of the manuscript. All authors contributed to the article and approved the submitted version.

## Funding

Medical writing support was provided by ClinicalThinking and funded by an alliance between Merck KGaA, Darmstadt, Germany and Pfizer.

## Conflict of Interest

KF has received honoraria from Bristol Myers Squibb, Eisai, Novartis, Pfizer, and Roche and has provided a consulting or advisory role for Bristol Myers Squibb, Eisai, Novartis, Pfizer, and Roche. CL has received honoraria from Amgen, Bristol Myers Squibb, Incyte, Merck & Co., Novartis, Pfizer, Pierre Fabre, and Roche; reports serving as a consultant or advisor for Amgen, Bristol Myers Squibb, Merck & Co., Novartis, and Roche; is a member of a speakers’ bureau for Amgen, Bristol Myers Squibb, Novartis, and Roche; has received research funding from Bristol Myers Squibb and Roche; has received reimbursement for travel and accommodation expenses from Bristol Myers Squibb; and has other relationships with Avantis Medical Systems. GG has received honoraria from and provided a consulting or advisory role for Bayer, Eisai, Novartis, Pfizer, and PharmaMar and received research funding and reimbursement for travel and accommodation expenses from PharmaMar.

The remaining authors declare that the research was conducted in the absence of any commercial or financial relationships that could be construed as a potential conflict of interest.

## Abbreviations

CK, cytokeratin; CT, computed tomography; ECOG PS, Eastern Cooperative Oncology Group performance status; MCC, Merkel cell carcinoma; mMCC, metastatic Merkel cell carcinoma; MRI, magnetic resonance imaging; OS, overall survival; PD, progressive disease; PD1, programmed death 1; PD-L1, programmed death ligand 1; PET, positron emission tomography; Q2W, every 2 weeks; RECIST 1.1, Response Evaluation Criteria in Solid Tumors version 1.1; SRS, stereotactic radiosurgery.
